# Politics is making us sick: The negative impact of political engagement on public health during the Trump administration

**DOI:** 10.1371/journal.pone.0262022

**Published:** 2022-01-14

**Authors:** Kevin B. Smith

**Affiliations:** Department of Political Science, University of Nebraska-Lincoln, Lincoln, Nebraska, United States of America; University of California San Francisco, UNITED STATES

## Abstract

**Objectives:**

To quantify the effect of politics on the physical, psychological, and social health of American adults during the four-year span of the Trump administration.

**Methods:**

A previously validated politics and health scale was used to compare health markers in nationally representative surveys administered to separate samples in March 2017 (N = 800) and October 2020 (N = 700). Participants in the 2020 survey were re-sampled approximately two weeks after the 2020 election and health markers were compared to their pre-election baselines.

**Results:**

Large numbers of Americans reported politics takes a significant toll on a range of health markers—everything from stress, loss of sleep, or suicidal thoughts to an inability to stop thinking about politics and making intemperate social media posts. The proportion of Americans reporting these effects stayed stable or slightly increased between the spring of 2017 and the fall of 2020 prior to the presidential election. Deterioration in measures of physical health became detectably worse in the wake of the 2020 election. Those who were young, politically interested, politically engaged, or on the political left were more likely to report negative effects.

**Conclusions:**

Politics is a pervasive and largely unavoidable source of chronic stress that exacted significant health costs for large numbers of American adults between 2017 and 2020. The 2020 election did little to alleviate those effects and quite likely exacerbated them.

## Introduction

Political polarization significantly worsened in the United States during the administration of President Donald J. Trump. Indeed, polls suggest that during his term partisan discord escalated to the point where opposing political camps disagreed not just on policy and governing preferences but even on “basic facts” [1]. Those deepening divisions almost certainly exacerbated a pre-existing tendency for politics to take a toll on the physical, psychological and social health of Americans. Between Trump’s 2016 election victory and his 2020 re-election campaign psychotherapists reported a significant jump in patients reporting politics negatively affecting their mental health [2], the American Psychological Association identified politics as a major source of stress for American adults [3], and there were sizeable increases in rates of depression, anxiety, loss of sleep, and emotional reactivity among groups with high levels of opposition to President Trump such as Democrats, racial minorities and students [4–6].

The possibility that political engagement may have serious public health consequences and that those consequences are at historically high levels motivates the key objectives of the present study: To assess how political engagement affects physical, psychological and social health among adults in the United States, to establish whether those health impacts increased or decreased across the period of the Trump administration, and to isolate the effect, if any, of the 2020 election and its associated shift in partisan fortunes on those same dimensions of health. These aims are pursued in two studies. The first uses a survey taken shortly after Trump’s inauguration in March 2017 (N = 800) and a second taken approximately two weeks prior to the November 2020 election (N = 700). Both surveys were designed to be nationally representative of the adult population in the United States and include an identical 32-item political health battery developed and psychometrically validated by Smith et al [7]. These two surveys thus allow a statistical analysis of change in these measures between the beginning and end of the Trump administration. The second study is based on a follow-up survey administered to the 2020 sample approximately two weeks after the election, using the same health battery (N~600 for subjects completing both waves of the 2020 survey). This allows a repeated measures, within-subjects research design to examine whether the perceived health costs of political engagement changed as a result of the election.

Results suggest that a large numbers of adults—depending on the health item, estimates run from tens of millions to more than a hundred million—attribute a range of significant physical, psychological and social health costs to politics, that those numbers stayed high and in some cases almost certainly increased over the course of the Trump administration, and that the 2020 election and its aftermath increased rather than decreased those negative health impacts. While it is a fairly universal phenomenon, this research found the negative toll politics takes on health is consistently correlated with being younger, identifying with the Democratic Party, being actively engaged in politics, disdaining political opponents, and having lower levels of political knowledge.

### How politics can harm health

The mechanism by which politics can harm health is relatively well understood. Politics is a chronic stressor, saturating popular culture and permeating daily life through social media, various entertainment platforms and a 24-hour news cycle [8]. Politics shapes social networks and individual identity, and is a well-documented source of negative emotions that predict self-reports of decreased psychological and physical well-being [9]. Elections and their associated lengthy campaigns act as cyclical accelerants to what are already high levels of politically-sourced stress. The negative effects of politics on social well-being—be it through passive attention or active engagement—is documented by a number of existing studies. For example, a fifth of Americans report being targets of online harassment as a result of expressing political views, more than two-thirds report recent elections as a significant source of stress in their lives, supporters of losing candidates engage in more stress-related behaviors such as increased alcohol consumption, and greater exposure to political campaign ads also increases the odds of being diagnosed by a health care professional with a psychological health condition such as anxiety or depression [10–13]. In addition to being measurable attitudinally and behaviorally, politically-induced stress is detectable physiologically. For example, political engagement is reported to correlate with baseline levels of cortisol, witnessing political conflict increases skin conductance levels (i.e. activation of the sympathetic “fight or flight” nervous system), and supporters of losing presidential candidates experience a drop in testosterone levels [14–16].

The negative health implications of stress, especially chronic stress, for a range of psychological and physical conditions is well documented [17, 18]. The notion that politics as a chronic source of stress could exact a toll on public health not only seems entirely plausible, there is increasing evidence that it manifests itself clinically in a range of health conditions [2, 19, 20]. Although the mechanism by which politics could negatively affect the health of large numbers people seems well-understood, the larger public health ramifications are not. Relatively few studies have directly addressed the toll politics takes on public health and, to the best of my knowledge, no published studies have tracked the public health impacts of politics across significant time periods, nor analyzed how significant changes in the political environment might change health measures over the long-term.

## Data and methods

One of the few studies attempting to systematically assess the public health consequences of politics using nationally representative data is Smith et al [7], who proposed and psychometrically validated a 32-item survey specifically designed to measure the health-related impacts of political engagement. These items were modeled after the self-diagnostic instruments used by Alcoholics Anonymous (AA) and Gamblers Anonymous (GA) to assess the physical, psychological and social costs of addiction. For example, a question used on both the AA and GA batteries is: “Does gambling (your drinking) ever cause you to have difficulty sleeping.” The comparable item on the Smith et al. instrument is, “I have lost sleep because of politics” with response categories ranging from 1 = strongly disagree to 5 = strongly agree (see supplementary materials for wording on all 32-items). The full 32-item battery has four sub-scales: Physical health, emotional health, regretted behavior (essentially compulsive behavior, e.g., “I have vowed to spend less time on politics but failed to follow through”), and social and lifestyle health. Smith et al. also proposed a 10-item short form version of the general battery. Based on the numbers indicating agreement or strong agreement with their items, Smith et al. estimated 94 million Americans perceived politics as a significant source of stress, 44 million had lost sleep because of politics, nearly 30 million reported politics had harmed their physical health, and 11 million had suicidal thoughts because of politics. Those effects are similar to or even higher than comparable health impacts associated with alcohol in the National Longitudinal Study of Adolescent to Adult Health [21].

The Smith et al. survey was fielded in March 2017, just a few months after President Trump’s inauguration, and their data is publically accessible [22], thus providing a good baseline for examining the effects of politics on collective well-being across the span of the Trump administration. To examine such changes the same public opinion organization that administered the original survey (YouGov) was commissioned to field an identical health survey approximately two weeks before the November 2020 election. A follow-up survey was administered to the same sample approximately a month later (approximately two weeks after the 2020 election) to assess what, if any, impact the election had on these markers of politically-related public health. For the first 2020 survey YouGov interviewed 834 adults (age > = 19 years), who were matched to a sampling frame on gender, age, race and education using the 2017 American Community Survey, resulting in a representative sample of N = 700. The same 834 respondents were contacted for the second 2020 survey, which produced a weighted representative sample of N = 618 with responses in both waves (N’s reported in models below fluctuate based on response rates to the survey items). All surveys included a range of socio-demographic, trait and attitudinal variables. All measures and associated descriptive statistics are detailed in the online supplementary materials.

To assess the temporal aspect of politics and health, the full 32-item health battery, its four sub-scales, the 10-item short form, as well as all individual items were subject to difference of means tests (independent sample t-tests) between the 2017 and the pre-election 2020 samples (all scales had high internal consistency with Chronbach’s alphas > .83 and were constructed as item averages with a theoretical range of 1–5; see supplementary materials for details). A pooled regression was used to generate a point estimate of the average change in all these variables between 2017 and 2020 while controlling for political orientation, attitudes towards political opposites, gender, age, and race (choice of statistical controls was limited to variables available in both the 2017 and 2020 data sets). To assess the effects of the 2020 election on these same measures, pre- and post-election differences within subjects were analyzed using paired sample t-tests. A series of panel analyses using random effects estimators was also used to assess whether pre- and post-election differences in these health markers could be detected in the presences of a wider range of statistical controls that also included political interest, political knowledge, political participation, psychological resiliency and voting record (see supplementary materials for further details). This project was approved by the University of Nebraska-Lincoln Institutional Review Board (Project ID# 20601). All survey participants consented via an online statement.

## Results

The general pattern of political health impacts during the Trump administration is one of stability. Only three of the health survey’s 32 items showed significant mean differences between 2017 and 2020: politics causing fatigue, thinking about politics more than one would like, and politics creating problems in extended family (all p < .05, 2-tailed t-test). On these three items scores increased by approximately .30 points on a 5-point scale. Neither the full 32-item battery or any of its four subscales showed any significant differences between 2017 and 2020. Full results for all analyses are reported in the supplementary materials.

**Fig 1:**
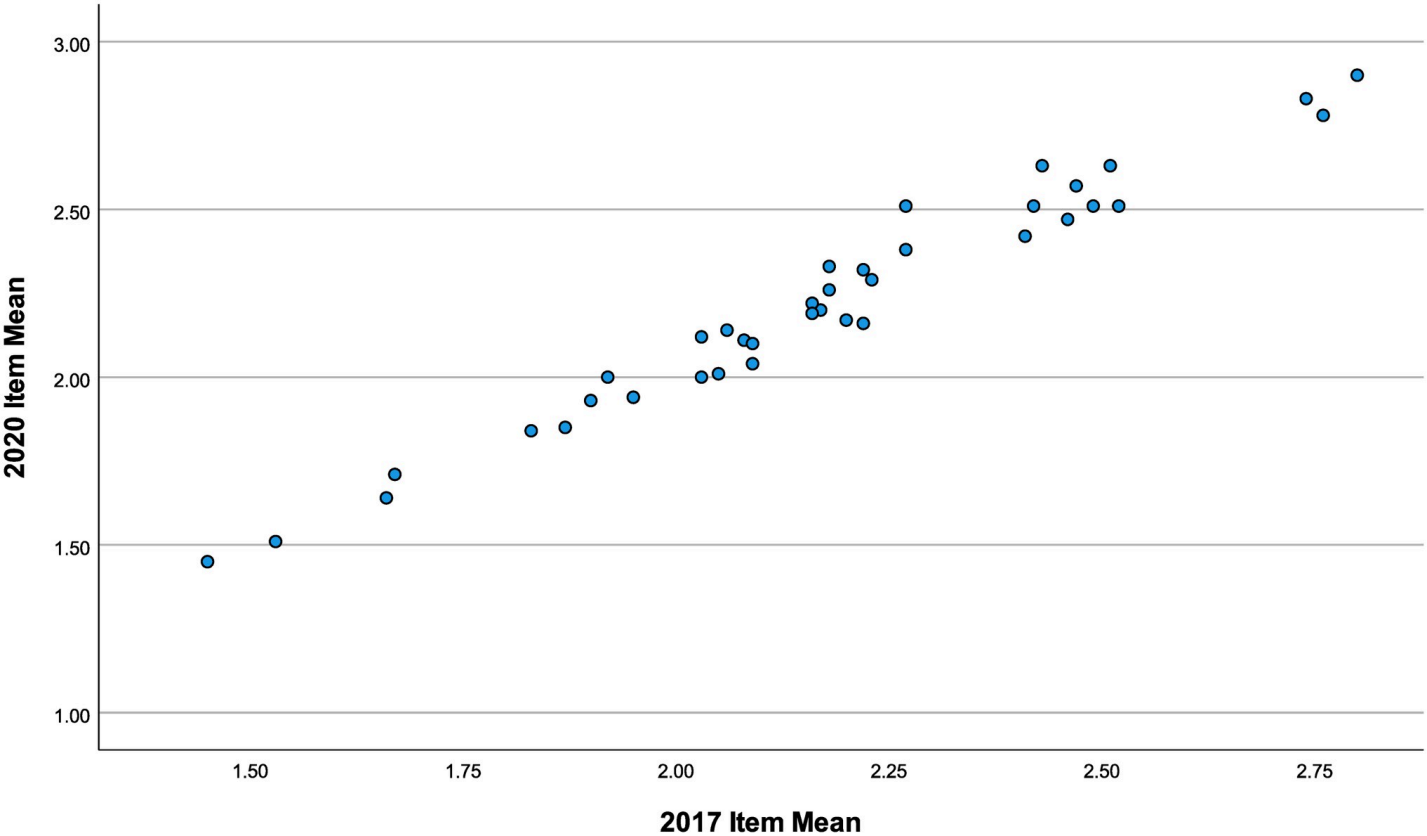
Mean politically-related health scores 2017–2020. Relationship between mean scores on all 32-items of Smith et al (2019) political costs health battery between 2017 and 2019.

A sense of the consistency of self-reported health impacts from two samples bookending the Trump administration is captured in a scatterplot (Fig 1) where mean scores for all 32 items for 2017 (x-axis) are plotted against their 2020 counterparts (y-axis). It shows little change across the entire range of health impacts captured by the survey. The correlation between the 2017 and 2020 means is .98 (p < .01). Again, this suggests the high levels of politically-related health impacts described by Smith et al. endured for the entire length of the Trump administration.

Pooling the two samples and running regression models that include a dummy variable to indicate sample year (1 = 2020, 0 = 2017) allows for a test of differences on the health scales in the presence of a range of statistical controls (see supplementary materials for full details on control variables). The coefficient for the dummy variable indicates the mean difference between 2017 and 2020 while accounting for these controls. Table 1 shows the results for the full 32-item battery and all associated subscales. As can be seen, these analyses do detect statistically significant increases in the physical health scale and the short form (10 item) general scale. Higher numbers indicate greater health impacts, and the dummy variable coefficient estimates for these two scales an average increase of ~0.10 points between 2017 and 2020 on these scales. Though significant, these effect sizes are so small they should be interpreted with caution. Especially given that in all the other models the estimated 2017–2020 differences were statistically indistinguishable from zero. These mostly null findings reinforce the general inference taken from the bivariate statistical tests: there simply was not much in the way of detectable differences in self-reported physical, psychological and social health costs attributed to politics between the 2017 and 2020 samples. The statistical detectable differences that were observed suggested politics was taking a slightly higher toll on public health in the fall of 2020 compared to the spring of 2017, but even here the differences were fairly modest. In short, lots of things changed between 2017 and 2020 but based on this data the negative health consequences attributed to politics remained remarkably stable.

**Table 1:** Regression models for combined 2017–2020 sample.

*Predictor/health scale*	*Full Scale*	*Physical Health Scale*	*Compulsion Scale*	*Social Lifestyle*	*Emotional Health Scale*	*10-item Short Scale*
***Constant***	1.739* (0.098)	1.63 (0.11)	1.701* (0.101)	1.915* (0.104)	1.676* (0.108)	1.378* (0.112)
***Partisanship***	-0.13* (0.025	-.23* (0.03)	-0.123* (0.026)	-0.106* (0.026)	-0.126* (0.028)	-0.202* (0.029)
***Political Interest***	0.148* (0.021)	.177* (0.25)	0.156* (0.022)	0.079* (0.022)	0.183* (0.023)	0.249* (0.024)
***Political Opposites***	0.041* (0.007)	0.50* (0.01)	0.028* (0.007)	0.024* (0.007)	0.071* (0.008)	0.078* (0.008)
***Gender***	0.09* (0.039)	-0.01 (0.04)	0.149* (0.04)	0.091* (0.041)	0.032 (0.043)	-0.005* (0.044)
***2020 Dummy***	0.027 (0.039)	0.09* (0.04)	0.035 (0.041)	0.021 (0.041)	0.021 (0.043)	0.123* (0.045)
***Black***	-0.22* (0.062)	-.30* (0.07)	-0.174* (0.064)	-0.187* (0.065)	-0.276* (0.069)	-0.291* (0.071)
***Age***	-0.01* (0.001)	-0.01* (0.001)	-0.008* (0.001)	-0.011* (0.001)	-0.011* (0.001)	-0.01* (0.001)
***N***	1327	1393	1381	1383	1381	1368
***F***	32.13	36.38	22.81	19.1	40.73	53.2
***Adj. R-2***	0.14	0.15	0.10	0.08	0.17	0.21

Unstandardized coefficient (standard error) reported,

* = p < .05.

For full description of variables and descriptive statistics on all variables see supplementary materials.

Though restricted to a more limited set of statistical controls than those used by Smith et al, the models reported in Table 1 largely mirror their findings in that those on the political left (Democrat Party identifiers), the young, the politically interested, and people who view those they politically disagree with as more close-minded and less truthful, were more likely to attribute negative health effects to politics. Generally speaking, males were more likely to report these effects, though the effect sizes here were small and inconsistent across models. Blacks were consistently less likely to report these negative effects. To assess whether there were significant differences between 2017 and 2020 within subgroups, a series of models were run using interaction terms (i.e., 2020 dummy X trait). These models are reported in S4 Table in the supplementary materials. The only interaction term that consistently reached statistical significance was for the interaction between the 2020 dummy and partisan identification. All else being equal, these models suggested that Republican Party identifiers were scoring ~.12 lower on the health impact scales in 2020 compared to 2017. This suggests that having more than three years of President Trump in office was associated with Republicans reporting lower health impacts compared to Democrats. Though significant, the difference is again relatively small. Similar interaction terms for age, race, gender and political interest were mostly insignificant (see table in supplementary materials for full details). Generally speaking, then, differences on the health measures between the 2017 and 2020 samples were small. But what about the 2020 election itself? Was the shift in partisan control of the White House correlated in any way with how people saw politics affecting their health? If so, was this change general or concentrated among those in particular groups or with certain individual-level traits? The second study presented here addresses those questions by examining individual differences in responses on the same health scales pre- and post-election. These within-individual changes over roughly a month bifurcated by the election were somewhat more pronounced than those estimated using comparisons of the October 2020 and March 2017 samples.

Paired sample t-tests were conducted on all individual health scale items, as well as the full and sub-scales and the short form general scale. Ten of the 32 individual items showed statistically significant pre- and post-election differences. Two of these items—“spending more time on political websites that I should” and “differences in political views have created problems for me in immediate family”—showed modest decreases (~.15 points, p < .05, two-tailed paired sample t-test). This suggests the election may have reduced, at least somewhat, the perceived impact of a few sources of politically-related stress. These, however, were the exception. Interestingly, statistically significant impacts were concentrated in the items that make up the physical health scale—five of the six measures constituting this scale indicated significant increases (i.e. respondents indicated a greater toll on their health). This is the same scale where a statistically significant increase was detected between 2017 and 2020. Compared to their pre-election baseline, post-election Americans indicated they were more stressed because of politics, more likely to be depressed because a favored candidate lost, were feeling more fatigued because of politics, were more likely to report losing sleep because of politics, and were more likely to say politics had harmed their physical health generally. The average increase was ~.22, which for those items represents roughly an increase of a quarter of a standard deviation in a one month span. In addition to the differences in individual items, the 10-item short form scale, the six-item physical impact health scale, and the eight-item emotional health impact scale all showed statistically significant pre- and post-election increases (all results reported in supplementary materials).

The significant increases associated with the physical health impact scale largely held up in panel analyses that controlled for a range of demographic and political variables (see Table 2). In these models the coefficient for the pre- and post-election dummy captures the mean change in the relevant health item while controlling for all other variables in the model. The pre- and post-election dummy variable for the physical health impact scale indicated that, all else equal, the mean score for the average American post-election was approximately .25 of a point higher compared to the pre-election baseline. Again, though, the predominant finding was one of relatively little change, though any statistically significant differences that were detected pointed towards post-election health being worse compared to pre-election health. In addition to the fairly substantive effect detected in the physical health scale, the emotional and short-form scales showed detectable post-election shifts albeit by much smaller increments (~.10). The pre- and post-election dummy was insignificant in the remaining three models, indicating no change on these scales.

**Table 2:** Panel analysis of pre/post 2020 election political health scales.

*Predictor/Health Scale*	*Full Scale*	*Physical Health Scale*	*Emotional Scale*	*Compulsion Scale*	*Social Scale*	*Short Scale*
***Age***	-0.01* (0.001)	-0.01* (0.002)	-.01* (.00)	-0.003* (0.001)	-0.01* (0.001)	-0.01* (0.001)
***Male***	0.09* (0.05)	-0.07 (0.05)	-.003 (.04)	0.19* (0.06)	0.15* (0.05)	-0.04 (0.06)
***Black***	-0.29* (0.08)	-0.34* (0.08)	-.39* (.09)	-0.19* (0.09)	-0.25* (0.08)	-0.45* (0.09)
***Partisanship***	-0.02 (0.04)	-0.06 (0.04)	-.05 (.05)	-0.03 (0.05)	0.03 (0.04)	-0.06 (0.05)
***Negative Partisanship***	0.25* (0.03)	0.29* (0.04)	.35* (.04)	0.20* (0.04)	0.13* (0.03)	0.37* (0.04)
***Resiliency Score***	-0.30* (0.03)	-0.37* (0.04)	-.29* (.04)	-0.31* (0.04)	-0.27* (0.04)	-0.33* (0.04)
***Political Interest***	-0.18* (0.03)	-0.23* (0.04)	-.15* (.04)	-0.17* (0.04)	-0.16* (0.04)	-0.24* (0.04)
***Political Knowledge***	-0.04* (0.01)	-0.03** (0.02)	-.01 (.02)	-0.06* (0.02)	-0.11* (0.02)	0.01 (0.02)
***Political Participation***	0.14* (0.02)	0.17* (0.02)	.13* (.02)	0.12* (0.02)	0.15* (0.02)	0.15* (0.02)
***Voted for Trump***	-0.11* (0.06)	-0.28* (0.08)	-.05 (.07)	-0.02 (0.07)	-0.14 (0.07)	-0.16* (0.07)
***Election Dummy***	0.04 (0.03)	0.24* (0.04)	.09* (.03)	-0.01 (0.03)	-0.03 (0.03)	0.13* (0.03)
***Constant***	3.33* (0.20)	3.82*** (0.2)	3.18* (.22)	3.31*** (0.23)	3.35*** (0.22)	3.53*** (0.22)
***N***	938	1,047	1,031	1,020	1,016	1,011
***F Statistic***	515*	535.46*	459*	309*	345*	599*
***Adjusted R^2^***	0.35	0.33	.30	0.23	0.25	0.37

Unstandardized coefficient (standard error) reported,

*p < .05 (2-tailed t-test).

These are random error estimators—see supplementary materials for statistical details.

The 2020 sample allowed a wider choice of statistical controls than the results reported in Table 1, though the expanded models reported in Table 2 tended to reinforce the main takeaways from the earlier analyses. Key Tables 1 and 2 differences in model specification are that the latter includes independently validated measures of negative partisanship (i.e. attitudes toward an opposing political party) [23] and psychological resiliency [24]. Similar to the simpler measure of “political opposites” used by Smith et al, the more sophisticated negative partisanship scale is consistently associated with higher levels of self-reported health impacts. Psychological resiliency—the ability to get past stressful events and “bounce back” from hard times—has the opposite effect. Again, the young, politically interested, and more politically engaged (i.e. higher levels of political participation) were more likely to report negative health impacts from politics. Being more informed, however, seemed to reduce the toll of politics. The negative coefficient for political knowledge (a test of civic knowledge developed by Pew Research) suggests that people who know more about politics and how the political system operates are somewhat less likely to report politics exacts a negative costs to their health.

There are two perhaps surprising results from the models reported in Table 2. First is the consistent finding that Blacks are again less likely to report these negative health costs—this is surprising in the sense that previous studies have suggested minorities may be particularly vulnerable to the stresses produced by politics [18]. The finding, however, is consistent across all the analyses reported here and are also consistent with those reported by Smith et al. The effect size is not huge, ~.30 compared to non-Blacks, but it is persistent. The second somewhat surprising finding is that people who reported voting for Trump in the 2020 election reported fewer negative health effects from politics. This seems inconsistent with the fact that their favored candidate lost the election. This, though, may simply be a function of the highly unusual aftermath of the 2020 election—many Trump supporters continued to believe their candidate would serve a second term in spite of overwhelming evidence to the contrary.

A series of follow-up models were run using interaction terms to assess whether an impact of the election on health might be mediated by a range of individual-level traits. The results of these analyses are reported in S8 Table of the supplementary materials. These do little to alter the key inferences taken from Table 2; even when statistically significant the effect sizes associated with the interaction terms were substantively small. For example, interaction between the election dummy and level of political knowledge was significant in all but one model, but the associated coefficient for this term was consistently around .04. This suggests that following the election someone with an average political knowledge score (4.03 on a 7-point scale) was reporting an increase of approximately .16 on the health scales compared to their pre-election scores. This does indeed suggest that those with more political knowledge—who tended to score lower on the health impact scales pre-election—saw politics as taking a greater toll on their health post-election. That estimated increase, though, is fairly marginal, amounting to roughly a fifth of a standard deviation. Most interaction terms failed to suggest even these sort of modest changes—the majority were statistically insignificant.

## Discussion

The central takeaway of the analyses is that virtually every health issue captured by the Smith et al. political health impact scales either stayed stable or increased across the four-year course of the Trump administration, and that the 2020 election was not associated with any big substantive change in this pattern. While there were some detectable health impacts associated with the 2020 election, these were fairly modest. What is the public health relevance of these findings? First and foremost is the fact that huge numbers of Americans clearly and consistently perceive politics as exacting a chronic negative toll on their health. Based on the 2019–20 Census Bureau population estimates, the resident population of the United States included approximately 255 million adults at the time of the 2020 survey. Based on that number, the findings from the pre-election survey suggest that somewhere between a fifth and a third of adults—roughly 50 to 85 million people—blame politics for causing fatigue, lost sleep, feelings of anger, loss of temper, as well as triggering compulsive behaviors (e.g. difficulty in stopping thinking about politics and consuming political information), and difficulties in impulse control (e.g. posting social media comments they later regretted; these estimates calculated using the percent agreeing or strongly agreeing with relevant survey items). A quarter of Americans reported seriously considering moving because of politics, and an estimated 40 percent—more than 100 million—consistently identify politics as a significant source of stress in their lives. Astonishingly, all three surveys consistently indicate that around five percent of adults report having suicidal thoughts because of politics—that’s an estimated 12 million people. Overall, these findings could hardly be more supportive of previous research arguing that more attention should be paid to the link between politics and health [25].

Second, these numbers do not seem to reflect the short-term post-election effect of an unpopular president taking office, nor do they seem to be the product of measurement error associated with a single sampling frame or an unreliable instrument. Scores were either stable or slightly increased across nearly a four-year time span in three nationally representative surveys (pre- and post-election scores on the 32-item scale correlated at r = .72). Third, having the same unpopular president lose re-election apparently did little to alleviate the suffering millions of Americans attributed to politics. It is possible, of course, that this is due primarily to the unusual aftermath of the 2020 election, which saw a sitting president not only refusing to concede an election loss but actively promoted baseless claims of election fraud. The data employed in this study, however, cannot speak directly to this possibility. It is also possible that these political health markers were influenced in some way by the unique circumstances of COVID-19 and partisan disagreement over the government’s response to the pandemic. The fact that these markers looked so similar in pre-pandemic 2017 and in October 2020 when the pandemic was at its height, however, suggests any such effects were minimal. As mentioned above, there is considerable individual-level variation in health scores (e.g. Trump voters tend to have lower scores than non-Trump voters), but the consistent, aggregate picture is that large proportions of the electorate see politics exacting a negative toll on their health, perceptions that changed little from 2017 to 2020 and, if anything, slightly worsened within individuals after the 2020 election.

The results here indicate that Americans see politics as significantly degrading their physical, psychological and social health and that, if anything, the most recent presidential election worsened these effects. Addressing this problem in any meaningful sense clearly presents a challenge. Traditionally political engagement has been conceived of as a public good, not as a threat to public health. A healthy and functioning democracy, after all, requires citizen engagement and participation. An obvious way to minimize a threat to public health is to minimize exposure, but to do so in this case seems civically irresponsible as it would prescribe not being an attentive and informed citizen. Following that course of action might increase the health of the public, but risks decreasing the democratic health of the polity. It seems likely that a political climate less fractious and polarized than that from 2016 to 2020 will reduce these health impacts naturally, something future research should investigate. High levels of conflict and polarization, though, seem likely to be characteristic of the American political system for the foreseeable future. Given that, how is it possible to maximize public engagement while minimizing the toll on the physical, psychological and emotional health of American adults?

Part of the answer may be found in the consistent findings of who is most likely to report that politics has a negative impact on their health—the young, left-leaning (Democratic identifiers), politically interested, and politically engaged—as well as the consistently prophylactic effect of a variable that can be manipulated, i.e. political knowledge. To be sure, higher levels of civic knowledge are unlikely to “cure” the problem. The models reported in Table 2 estimate someone with full marks on the knowledge is likely to drop by ~.70 of a point on a 1–5 health scale. Still, increasing understanding of the political system among the young and politically engaged is unlikely to hurt, and may very well help.

## Supporting information

S1 Table Descriptive statistics and mean differences in health survey items 2017 vs. 2020. (DOCX)

S2 Table Health scale and sub-scale descriptives and differences 2017–2020. (DOCX)

S3 Table Measures, means and standard deviations of control variables used in 2017–2020 pooled regression. (DOCX)

S4 Table Full regression models with interaction terms for combined 2017–2020 sample. (DOCX)

S5 Table Pre/post 2020 election mean differences in health survey items. (DOCX)

S6 Table Health scale and sub-scale pre/post 2020 election differences. (DOCX)

S7 Table Measures, means and standard deviations of control variables used in pre/post election panel analysis. (DOCX)

S8 Table Panel analysis of pre/post 2020 election health scores with interaction terms. (DOCX)

S9 Table Test of fixed versus random effects estimators. (DOCX)

## References

[1] Dimock, Michael and John Gramlich. 2021. “How American Changed During Donald Trump’s Presidency,” Pew Research Center. https://www.pewresearch.org/2021/01/29/how-america-changed-during-donald-trumps-presidency/

[2] Farber Barry A. 2018. “‘Clowns to the left of me, Jokers to the right’: Politics and Psychotherapy, 2018”. Journal of Clinical Psychology. 74: 714–721. doi: 10.1002/jclp.22600 29543330

[3] American Psychological Association. Stress in America: coping with change. February 15, 2017 (http://www.apa.org/news/press/releases/stress/2016/coping-with-change.PDF)

[4] Krupenkin M., Rothschild D., Hill S., & Yom-Tov E. (2019). President Trump stress disorder: partisanship, ethnicity, and expressive reporting of mental distress after the 2016 election. Sage open, 9(1), 2158244019830865.

[5] Roche M. J., & Jacobson N. C. (2019). Elections Have Consequences for Student Mental Health: An Accidental Daily Diary Study. *Psychological Reports*, 122(2), 451–464. doi: 10.1177/0033294118767365 29621944

[6] Neupert S. D., Bellingtier J. A., & Smith E. L. (2019). Emotional reactivity changes to daily stressors surrounding the 2016 U.S. presidential election. *Current Psychology*.

[7] Smith K. B., Hibbing M. V., & Hibbing J. R. (2019). Friends, relatives, sanity, and health: The costs of politics. *PloS one*, 14(9), e0221870. doi: 10.1371/journal.pone.0221870 31553726 PMC6760758

[8] Ford Brett and Feinberg Matthew. 2020. “Coping with Politics: The Benefits and Costs of Emotion Regulation.” *Current Opinion in Behavioral Sciences*. 34: 123–128.

[9] Feinberg Matthew, Ford Brett, Thai Sabrina, Gatchpasian Arasteh and Lassetter Bethany. 2020. “The Political is Personal: Daily Politics as a Chronic Stressor.” *PsyArXiv*. doi: 10.31234/osf.io/hdz97 36689389

[10] Vogels, Emily. (Jan. 13, 2021). “The State of Online Harassment.” Pew Research Center. https://www.pewresearch.org/internet/2021/01/13/the-state-of-online-harassment/?utm_source=Pew+Research+Center&utm_campaign=7e5d769390-EMAIL_CAMPAIGN_2021_02_18_09_30&utm_medium=email&utm_term=0_3e953b9b70-7e5d769390-400856725

[11] American Psychological Association. (October 7, 2020). “2020 Presidential Election a Source of Significant Stress for More Americans than 2016 Presidential Race.” https://www.apa.org/news/press/releases/2020/10/election-stress

[12] Musse, Isabel and Schneider, Rodrigo, The Effect of Presidential Election Outcomes on Alcohol Drinking (July 28, 2020). SSRN: https://ssrn.com/abstract=3662663 or 10.2139/ssrn.3662663

[13] Niederdeppe J., Avery R. J., Liu J., Gollust S. E., Baum L., Barry C. L.,… et al. (2021). Exposure to Televised Political Campaign Advertisements Aired in the United States 2015–2016 Election Cycle and Psychological Distress. Social Science & Medicine, 113898. doi: 10.1016/j.socscimed.2021.113898 33848716

[14] French Jeffrey A, Smith Kevin B., Alford John R., et al. 2014. “Cortisol and Politics: Variance in Voting Behavior is Predicted by Baseline Cortisol Levels.” *Physiology & Behavior*. 133: 61–67. doi: 10.1016/j.physbeh.2014.05.004 24835544 PMC4120245

[15] Mutz Diana and Reeves Byron. 2005. “The New Videomalaise: Effects of Televised Incivility on Political Trust.” *American Political Science Review*. 99: 1–15.

[16] Stanton Steven J., Beehner Jacinta, Saini Ekjyot, Kuhn Cynthia and Kevin LaBar. 2009. “Dominance, Politics, and Physiology: Voters’ Testosterone Changes on the Night of the 2008 United States Presidential Eleciton.” *PLoS One*. doi: 10.1371/journal.pone.0007543 19844583 PMC2760760

[17] Schneiderman N., Ironson G., & Siegel S. D. (2005). Stress and health: psychological, behavioral, and biological determinants. Annual review of clinical psychology, 1. 10.1146/annurev.clinpsy.1.102803.144141 PMC2568977 17716101

[18] Sapolsky, R. M. (2004). *Why zebras don’t get ulcers*: *The acclaimed guide to stress*, *stress-related diseases*, *and coping*. Holt paperbacks.

[19] Blakely T.S., Kennedy B.P., and Kawachi I. Socioeconomic Inequality in Voting Participation and Self-Rated Health. *Am J Public Health*. 2001. 91(1): 99–104. doi: 10.2105/ajph.91.1.99 . 11189832 PMC1446487

[20] Hagan M., Sladek M., Luecken L. and Doane L. Event-Related Clinical Distress in College Students. *J Am College Health*. 2018. Available from: Https://www.tandfonline.com/doi/full/10.1080/07448481.2018.1515763 30346876 10.1080/07448481.2018.1515763

[21] Harris, K. The National Longitudinal Study of Adolescent to Adult Health, Waves I & II. 2009. Database: Data Sharing for Demographic Research. https://www.icpsr.umich.edu/icpsrweb/DSDR/studies/27021/version/9

[22] Smith, Kevin, 2019, "Replication Data for Friends, Sanity, Health PLoS One", 10.7910/DVN/WCPGAU, Harvard Dataverse, V1, UNF:6:4+9XbIMDr4xzUJE6Yfd0pg==

[23] Bankert A. 2020. Negative and Positive Partisanship in the 2016 U.S. Presidential Elections. *Polit Behav* doi: 10.1007/s11109-020-09599-1

[24] Smith B.W., Dalen J., Wiggins K., Tooley E., Christopher P. & Bernard J. (2008). The brief resilience scale: assessing the ability to bounce back. International Journal of Behavioral Medicine, 15, 194–200. doi: 10.1080/10705500802222972 18696313

[25] Pacheco J. and Fletcher J. 2015. “Incorporating Health into Studies of Political Behavior: Evidence for Turnout and Partisanship.” *Political Research Quarterly*. 68: 104–116. doi: 10.1177/1065912914563548 30008544 PMC6042216

