# Supplementary material for: Politics is making us sick: The negative impact of political engagement on public health during the Trump administration
Source: PLoS One. 2022 Jan 14;17(1):e0262022. doi: 10.1371/journal.pone.0262022 (PMC8759681; doi:10.1371/journal.pone.0262022)
Supplement: S1 Table — (DOCX) [file pone.0262022.s001.docx]

**Table S1:** Descriptive Statistics and Mean Differences in Health Survey Items 2017 vs. 2020

| **Physical Health Scale Items** | 2017 Mean | 2017 Std. Deviation | 2020 Mean | 2020 Std. Deviation | Diff means t-test |
| --- | --- | --- | --- | --- | --- |
| Politics has caused me to be stressed# | 2.69 | 1.353 | 2.82 | 1.408 | 1.89 |
| I have become depressed when a preferred candidate lost# | 2.43 | 1.277 | 2.4 | 1.275 | -0.54 |
| Politics has caused me to be fatigued# | 2.22 | 1.174 | 2.46 | 1.386 | 3.5* |
| I have lost sleep because of politics | 2.05 | 1.169 | 2.08 | 1.287 | 0.58 |
| Politics has adversely affected my physical health, even if only a little | 1.92 | 1.038 | 1.98 | 1.188 | 1.06 |
| Politics has caused me to be suicidal | 1.51 | 0.933 | 1.49 | 0.981 | -0.55 |
| **Emotional Health Scale Individual Items** |  |  |  |  |  |
| Exposure to media outlets promoting views contrary to mine can drive me crazy# | 2.75 | 1.28 | 2.8 | 1.291 | 0.75 |
| I have lost my temper as a result of politics# | 2.56 | 1.335 | 2.48 | 1.356 | -1.2 |
| Politics has led me to hate some people# | 2.47 | 1.263 | 2.48 | 1.359 | 0.11 |
| Politics has caused me to think seriously about moving# | 2.29 | 1.296 | 2.37 | 1.45 | 1.18 |
| On occasion, I have regretted comments I made during a political discussion | 2.26 | 1.141 | 2.17 | 1.173 | -1.5 |
| I have secretly wished bad things on those who disagree with me politically | 2.11 | 1.123 | 2.04 | 1.157 | -1.2 |
| I sometimes feel guilty about the way I feel toward those who disagree with me | 2.24 | 1.072 | 2.22 | 1.16 | -0.42 |
| I have become annoyed when others are critical of my political views | 2.74 | 1.223 | 2.74 | 1.274 | 0.02 |
| **Compulsive Behavior Scale Individual Items** |  |  |  |  |  |
| I spend more time thinking about politics than I would like# | 2.42 | 1.246 | 2.65 | 1.334 | 3.4* |
| I care too much about who wins and loses in politics# | 2.53 | 1.15 | 2.58 | 1.242 | 0.79 |
| My life would be better if I didn't focus so much on politics | 2.43 | 1.092 | 2.51 | 1.208 | 1.33 |
| At times, I wish I would have restrained myself more in political conversations | 2.28 | 1.08 | 2.32 | 1.193 | 0.72 |
| I have posted or written things on-line that I later wished I hadn't | 2 | 1.133 | 2.06 | 1.246 | 0.98 |
| I have vowed to spend less time on politics but failed to follow through | 2.25 | 1.099 | 2.31 | 1.204 | 1.06 |
| I spend more time on political websites than I should | 2.02 | 1.073 | 2.15 | 1.216 | 2.11* |
| Politics has sometimes caused me to exercise bad judgment | 1.96 | 1.011 | 1.98 | 1.154 | 0.26 |
| My interest in politics has delayed me from completing an assignment, task, or job | 1.84 | 0.969 | 1.88 | 1.181 | 0.68 |
| After a major election or political event, there is sometimes a void in my life | 2.08 | 1.051 | 1.99 | 1.112 | -1.61 |
| **Social and Lifestyle Health Scale Individual Items** |  |  |  |  |  |
| Differences in political views have damaged a friendship I valued# | 2.25 | 1.174 | 2.29 | 1.271 | 0.68 |
| Differences in political views have created problems for me in my extended family | 2.16 | 1.139 | 2.32 | 1.337 | 2.43* |
| On occasion, politics has made my home life less pleasant | 2.11 | 1.142 | 2.14 | 1.281 | 0.47 |
| Differences in political views have created problems for me in my immediate family | 2.05 | 1.165 | 2.1 | 1.258 | 0.85 |
| Differences in political views have created problems for me at work | 1.88 | 0.968 | 1.91 | 1.13 | 0.49 |
| I have lost time from work or school because of politics | 1.69 | 0.929 | 1.74 | 1.057 | 0.85 |
| My political views have created financial problems for me | 1.69 | 0.911 | 1.67 | 1.008 | -0.37 |
| My political views have created legal problems for me | 1.59 | 0.904 | 1.55 | 0.985 | -0.85 |

# Item included in 10-item short form scale recommended by Smith et al (2019)

* difference between 2017 and 2020 mean significant at p < .05, independent samples t-test (2-tailed)

N=800 for 2017, N ~ 680 for 2020 items

Means used to produce scatterplot reported in Figure 1.
