# Supplementary material for: Politics is making us sick: The negative impact of political engagement on public health during the Trump administration
Source: PLoS One. 2022 Jan 14;17(1):e0262022. doi: 10.1371/journal.pone.0262022 (PMC8759681; doi:10.1371/journal.pone.0262022)
Supplement: S2 Table — (DOCX) [file pone.0262022.s002.docx]

**Table S2:** Health Scale and Sub-Scale Descriptives and Differences 2017-2020

|  | Mean 2017 | Std Dev 2017 | Chronbach’s Alpha 2017 | Mean 2020 | Std Dev  2020 | Chronbach’s Alpha 2020 | Diff Means t-test |
| --- | --- | --- | --- | --- | --- | --- | --- |
| Compulsive Behavior Scale | 2.18 | 0.74 | 0.87 | 2.21 | 0.83 | 0.88 | 0.71 |
| Social and Lifestyle Health Scale | 1.92 | 0.75 | 0.86 | 1.95 | 0.83 | 0.86 | 0.57 |
| Physical Health Scale | 2.13 | 0.88 | 0.85 | 2.18 | 0.95 | 0.85 | 1.01 |
| Emotional Health Scale | 2.42 | 0.84 | 0.85 | 2.41 | 0.87 | 0.83 | -0.3 |
| 10-time Short Form Scale | 2.46 | 0.88 | 0.88 | 2.53 | 0.93 | 0.88 | 1.43 |
| Full 32-item Scale | 2.17 | 0.72 | 0.95 | 2.18 | 0.80 | 0.95 | 0.29 |

N=800 for 2017, N ~ 680 for 2020 items. Difference of means test is an independent samples t-test (2-tailed), no scales showed significant differences between 2017 and 2020
