# Supplementary material for: Politics is making us sick: The negative impact of political engagement on public health during the Trump administration
Source: PLoS One. 2022 Jan 14;17(1):e0262022. doi: 10.1371/journal.pone.0262022 (PMC8759681; doi:10.1371/journal.pone.0262022)
Supplement: S3 Table — (DOCX) [file pone.0262022.s003.docx]

**Table S3**. Measures, Means and Standard Deviations of Control Variables Used in 2017-2020 Pooled Regression

| Variable | Measurement and coding | 2017 Mean | 2017 Std Dev | 2020  Mean | 2020 Std  Def | Diff means t-test |
| --- | --- | --- | --- | --- | --- | --- |
| Partisanship | 1=Dem, 0= Indep, 1=Rep | -0.12 | 0.77 | -0.06 | 0.80 | 1.4 |
| Political Interest | 4-item scale, 1= low, 4=high | 3.14 | 0.98 | 3.18 | 0.96 | 0.89 |
| Political Opposites | 3-item index of attitudes on political opposites (more informed, more close minded, less truthful) | 10.25 | 2.85 | 9.55 | 2.82 | -4.7* |
| male | 1 =male, 0 = female | 0.48 | 0.49 | 0.48 | 0.50 | 0.19 |
| black | 1 = black, 0= other | 0.11 | 0.32 | 0.11 | 0.32 | 0.09 |
| age | age in years | 47.92 | 17.0 | 47.72 | 17.64 | -0.22 |
| Valid N (listwise) |  | 779 |  | 549 |  |  |

* p < .05 (2-tailed t-test)
