# Supplementary material for: Politics is making us sick: The negative impact of political engagement on public health during the Trump administration
Source: PLoS One. 2022 Jan 14;17(1):e0262022. doi: 10.1371/journal.pone.0262022 (PMC8759681; doi:10.1371/journal.pone.0262022)
Supplement: S4 Table — (DOCX) [file pone.0262022.s004.docx]

**Table S4.** Full Regression Models With Interaction Terms For Combined 2017-2020 Sample

|  | Full 32 Item | Physical Health | Compulsion | Social Lifestyle | Emotional | 10-item short scale |
| --- | --- | --- | --- | --- | --- | --- |
| Constant | 1.749*  (0.11) | 1.666*  (.14) | 1.713*  (.12) | 1.952*  (.12) | 1.611*  (.13) | 1.299*  (.13) |
| Partisanship | -0.09*  (.03) | -0.191*  (.04) | -0.068*  (.04) | -0.085*  (.03) | -0.066  (.04) | -0.132*  (.04) |
| Political Interest | 0.152*  (.02) | 0.164*  (.03) | 0.166*  (.03) | 0.068*  (.02) | 0.205*  (.03) | 0.269*  (.03) |
| Political Opposites | 0.04*  (.01) | 0.051*  (.01) | 0.026*  (.01) | 0.025*  (.01) | 0.07*  (.00) | 0.077*  (.01) |
| Gender | 0.06  (.05) | 0.014  (.06) | 0.107*  (.05) | 0.102  (.054) | 0.022  (.06) | -0.01  (.06) |
| 2020 Dummy | 0.03  >16) | 0.031  (.18) | 0.042  (.16) | -0.069  (.17) | 0.193  (.18) | 0.339  (.18) |
| Black | -0.13  (.08) | -0.211*  (.10) | -0.113  (.08) | -0.102  (.08) | -0.141  (.09) | -0.133  (.09) |
| Age | -0.01*  (.00) | -0.012*  (.002) | -0.008*  (.00) | -0.011*  (.00) | -0.011*  (.00) | -0.01*  (.00) |
| Black X Year | -0.218  (.12) | -0.222  (.14) | -0.154  (.13) | -0.204  (.13) | -0.32*  (.13) | -0.385*  (.14) |
| Age X Year | 0.001  (.002) | 0.001  (.003) | 0.001  (.002) | 0.001  (.002) | 0.001  (.003) | 0  (.003) |
| Partisanship X Year | -0.108*  (.05) | -0.105  (.06) | -0.127*  (.05) | -0.046  (.05) | -0.147*  (.05) | -0.171*  (.06) |
| Gender X Year | 0.057  (.07) | -0.04  (.09) | 0.1  (.08) | -0.026  (.08) | 0.028  (.09) | 0.014  (.09) |
| Political interest X Year | -0.012  (.04) | 0.023  (.05) | -0.03  (.04) | 0.025  (.04) | -0.059  (.05) | -0.054  (.05) |
| N | 1327 | 1393 | 1381 | 1383 | 1381 | 1368 |
| F | 19.3* | 21.6* | 13.9* | 11.48 | 24.7* | 32.39 |
| Adj. R-2 | 0.15 | 0.15 | 0.10 | 0.08 | 0.17 | 0.21 |

Unstandardized coefficient (standard error) reported, * = p < .05.

Regressions conducted on the combined 2017/2020 samples. The 2020 dummy is 1=2020 sample and 0=2017 sample, and the coefficient for this variable represents the mean difference between the two samples while accounting for all other variables in the model.
