# Supplementary material for: Politics is making us sick: The negative impact of political engagement on public health during the Trump administration
Source: PLoS One. 2022 Jan 14;17(1):e0262022. doi: 10.1371/journal.pone.0262022 (PMC8759681; doi:10.1371/journal.pone.0262022)
Supplement: S5 Table — (DOCX) [file pone.0262022.s005.docx]

**Table S5:** Pre/Post 2020 Election Mean Differences in Health Survey Items

|  | Pre-Election Mean | Post-Election Mean | Diff means t-test |
| --- | --- | --- | --- |
| **Physical Health Scale Items** |  |  |  |
| Politics has caused me to be stressed | 2.88 | 3.36 | -9.12* |
| I have become depressed when a preferred candidate lost. | 2.53 | 2.65 | -2.43* |
| Politics has caused me to be fatigued. | 2.47 | 2.73 | -4.85* |
| I have lost sleep because of politics. | 2.15 | 2.35 | -3.763* |
| Politics has adversely affected my physical health, even if only a little. | 1.95 | 2.26 | -6.559* |
| Politics has caused me to be suicidal. | 1.47 | 1.53 | -0.848 |
| **Emotional Health Scale Individual Items** |  |  |  |
| Exposure to media outlets promoting views contrary to mine can drive me crazy. | 2.87 | 2.89 | -0.726 |
| I have lost my temper as a result of politics. | 2.5 | 2.49 | -0.073 |
| Politics has led me to hate some people. | 2.47 | 2.79 | -6.566* |
| Politics has caused me to think seriously about moving. | 2.4 | 2.42 | -1.17 |
| On occasion, I have regretted comments I made during a political discussion. | 2.13 | 2.19 | -1.093 |
| I have secretly wished bad things on those who disagree with me politically. | 2.09 | 2.18 | -2.132* |
| I sometimes feel guilty about the way I feel toward those who disagree with me. | 2.18 | 2.23 | -0.976 |
| I have become annoyed when others are critical of my political views. | 2.79 | 2.73 | 1.239 |
| **Compulsive Behavior Scale Individual Items** |  |  |  |
| I spend more time thinking about politics than I would like. | 2.64 | 2.61 | 0.717 |
| I care too much about who wins and loses in politics. | 2.65 | 2.63 | 0.355 |
| My life would be better if I didn't focus so much on politics. | 2.53 | 2.64 | -2.022* |
| At times, I wish I would have restrained myself more in political conversations. | 2.25 | 2.31 | -0.909 |
| I have posted or written things on-line that I later wished I hadn't. | 2.03 | 1.97 | 1.022 |
| I have vowed to spend less time on politics but failed to follow through. | 2.23 | 2.26 | -0.179 |
| I spend more time on political websites than I should. | 2.25 | 2.09 | 3.287* |
| Politics has sometimes caused me to exercise bad judgment. | 1.95 | 1.88 | 1.792 |
| My interest in politics has delayed me from completing an assignment, task, or job. | 1.82 | 1.83 | -0.936 |
| After a major election or political event, there is sometimes a void in my life. | 1.98 | 1.94 | 1.059 |
| **Social and Lifestyle Health Scale Individual Items** |  |  |  |
| Differences in political views have damaged a friendship I valued. | 2.38 | 2.46 | -1.611 |
| Differences in political views have created problems for me in my extended family. | 2.38 | 2.33 | 1.234 |
| On occasion, politics has made my home life less pleasant. | 2.04 | 2.06 | -0.623 |
| Differences in political views have created problems for me in my immediate family. | 2.14 | 1.99 | 3.286* |
| Differences in political views have created problems for me at work. | 1.84 | 1.84 | -0.096 |
| I have lost time from work or school because of politics. | 1.72 | 1.68 | 0.394 |
| My political views have created financial problems for me. | 1.66 | 1.59 | 1.763 |
| My political views have created legal problems for me. | 1.55 | 1.59 | -0.851 |

** P < .05, paired sample t-test (2-tailed)

Valid N (listwise) = 502.

Note: pre-election 2020 means may slightly differ from those reported in 2017-2020 comparison, this is because only those who responded to both pre- and post-election surveys are included here.
