# Supplementary material for: Politics is making us sick: The negative impact of political engagement on public health during the Trump administration
Source: PLoS One. 2022 Jan 14;17(1):e0262022. doi: 10.1371/journal.pone.0262022 (PMC8759681; doi:10.1371/journal.pone.0262022)
Supplement: S6 Table — (DOCX) [file pone.0262022.s006.docx]

**Table S6:** Health Scale and Sub-Scale Pre/Post 2020 Election Differences

| Health Scale | Pre-Election Mean | Post-Election Mean | t-test of mean differences |
| --- | --- | --- | --- |
| Physical Health Scale | 2.228 | 2.4997 | -7.203* |
| Social and Lifestyle Health Scale | 1.9362 | 1.8978 | 0.354 |
| Emotional Health Scale | 2.4267 | 2.5011 | -3.334* |
| Compulsive Behavior Scale | 2.2024 | 2.2234 | -0.078 |
| 10-item Short Form Scale | 2.5724 | 2.7368 | -4.656* |
| Full 32-item Scale | 2.1938 | 2.2427 | -1.61 |

* P < .05, paired sample t-test (2-tailed)
