# Supplementary material for: Politics is making us sick: The negative impact of political engagement on public health during the Trump administration
Source: PLoS One. 2022 Jan 14;17(1):e0262022. doi: 10.1371/journal.pone.0262022 (PMC8759681; doi:10.1371/journal.pone.0262022)
Supplement: S7 Table — (DOCX) [file pone.0262022.s007.docx]

**Table S7.** Measures, Means and Standard Deviations of Control Variables Used in Pre/Post Election Panel Analysis

| Variable | Measurement and coding | Mean/Stand Dev |
| --- | --- | --- |
| Partisanship | 1=Dem, 0= Indep, 1=Rep | -.04  (.80) |
| Political Interest | 4-item scale, 1= low, 4=high | 1.77  (.95) |
| Negative Partisanship | 1-4 score based on mean of 8 item Bankert (2016) negative partisanship scale | 2.5  (.81) |
| Resiliency Score | 1-5 score based on Smith et al (2008) brief resiliency score | 3.3  (.74) |
| Political Knowledge | 1-7 score based on correct answers to 7 questions | 4.03  (2.11) |
| Political Participation | 1-5 score based on number of ‘yes’ answers to 5 types of political participation | 1.22  (1.36) |
| Voted for Trump | 1=voted for Trump, 0=did not vote for Trump | .25  (.43) |
| male | 1 =male, 0 = female | 0.48 |
| black | 1 = black, 0= other | 0.11 |
| age | age in years | 47.92 |
