# Supplementary material for: Politics is making us sick: The negative impact of political engagement on public health during the Trump administration
Source: PLoS One. 2022 Jan 14;17(1):e0262022. doi: 10.1371/journal.pone.0262022 (PMC8759681; doi:10.1371/journal.pone.0262022)
Supplement: S8 Table — (DOCX) [file pone.0262022.s008.docx]

**Table S8**. Panel Analysis of Pre/Post 2020 Election Health Scores With Interaction Terms

|  | Full  Battery  (32 Item) | Health  (6 Item) | Emotional  (8 Item) | Compulsion  (10 Item) | Social  (8 Item) | Short Form (10 Item) |
| --- | --- | --- | --- | --- | --- | --- |
|  | | | | | | |
| Age | -0.01^*^  (0.002) | -0.01^*^  (0.002) | -0.01^*^  (0.002) | -0.005^*^  (0.002) | -0.01^*^  (0.002) | -0.01^*^  (0.002) |
|  |  |  |  |  |  |  |
| Male | 0.13^*^  (0.05) | 0.004  (0.07) | 0.06  (0.06) | 0.20^*^  (0.06) | 0.16^*^  (0.06) | 0.03  (0.06) |
|  |  |  |  |  |  |  |
| Black | -0.23^*^  (0.08) | -0.33^*^  (0.10) | -0.35^*^  (0.10) | -0.14  (0.10) | -0.22^*^  (0.09) | -0.43^*^  (0.10) |
|  |  |  |  |  |  |  |
| Partisanship | -0.03  (0.04) | -0.07  (0.05) | -0.04  (0.05) | -0.04  (0.05) | 0.04  (0.05) | -0.08  (0.05) |
|  |  |  |  |  |  |  |
| Negative Partisanship | 0.27^*^  (0.03) | 0.30^*^  (0.04) | 0.38^*^  (0.04) | 0.20^*^  (0.04) | 0.16^*^  (0.04) | 0.39^*^  (0.04) |
|  |  |  |  |  |  |  |
| Resiliency Score | -0.30^*^  (0.03) | -0.37^*^  (0.04) | -0.29^*^  (0.04) | -0.31^*^  (0.04) | -0.26^*^  (0.04) | -0.33^*^  (0.04) |
|  |  |  |  |  |  |  |
| Political Interest | -0.17^*^  (0.04) | -0.19^*^  (0.05) | -0.13^*^  (0.04) | -0.17^*^  (0.04) | -0.17^*^  (0.04) | -0.21^*^  (0.04) |
|  |  |  |  |  |  |  |
| Political Knowledge | -0.07^*^ | -0.05^*^  (0.02) | -0.02  (0.02) | -0.07^*^  (0.02) | -0.12^*^  (0.02) | -0.01  (0.02) |
|  | (0.02) |  |  |  |  |  |
|  |  |  |  |  |  |  |
| Political Participation | 0.14^*^  (0.02) | 0.17^*^  (0.03) | 0.12^*^  (0.02) | 0.13^*^  (0.02) | 0.15^*^  (0.02) | 0.14^*^  (0.02) |
|  |  |  |  |  |  |  |
|  |  |  |  |  |  |  |
| Voted for Trump | -0.12^*^  (0.06) | -0.29^*^  (0.08) | -0.07  (0.07) | -0.03  (0.07) | -0.16^*^  (0.07) | -0.17^*^  (0.07) |
|  |  |  |  |  |  |  |
|  |  |  |  |  |  |  |
| Post-Election Dummy | -0.20  (0.19) | 0.37  (0.24) | 0.10  (0.22) | -0.32  (0.20) | -0.21  (0.21) | 0.06  (0.22) |
|  |  |  |  |  |  |  |
|  |  |  |  |  |  |  |
| Election X Age | 0.004^*^  (0.002) | 0.001  (0.002) | 0.002  (0.002) | 0.003^*^  (0.002) | 0.004^*^  (0.002) | 0.003  (0.002) |
|  |  |  |  |  |  |  |
|  |  |  |  |  |  |  |
| Election X Black | -0.17^*^  (0.10) | -0.02  (0.12) | -0.10  (0.11) | -0.14  (0.10) | -0.06  (0.10) | -0.04  (0.11) |
|  |  |  |  |  |  |  |
|  |  |  |  |  |  |  |
| Election X Male | -0.11^*^  (0.06) | -0.17^*^  (0.08) | -0.16^*^  (0.07) | -0.02  (0.06) | -0.01  (0.06) | -0.16^*^  (0.07) |
|  |  |  |  |  |  |  |
|  |  |  |  |  |  |  |
| Election X Interest | -0.03  (0.04) | -0.09^*^  (0.05) | -0.06  (0.05) | 0.001  (0.04) | 0.01  (0.05) | -0.07  (0.05) |
|  |  |  |  |  |  |  |
| Election X Participation | -0.01  (0.02) | -0.02  (0.03) | 0.004  (0.03) | -0.02  (0.02) | -0.003  (0.02) | 0.002  (0.03) |
|  |  |  |  |  |  |  |
|  |  |  |  |  |  |  |
| Election X Knowledge | 0.05^*^  (0.02) | 0.04^*^  (0.02) | 0.04^*^  (0.02) | 0.04^*^  (0.02) | 0.03  (0.02) | 0.04^*^  (0.02) |
|  |  |  |  |  |  |  |
|  |  |  |  |  |  |  |
| Election X Negative Partisanship | -0.03  (0.04) | -0.03  (0.05) | -0.06  (0.04) | -0.01  (0.04) | -0.06  (0.04) | -0.03  (0.04) |
|  |  |  |  |  |  |  |
| Election X Partisanship | 0.01  (0.04) | 0.03  (0.05) | -0.02  (0.04) | 0.05  (0.04) | -0.01  (0.04) | 0.05  (0.04) |
|  |  |  |  |  |  |  |
|  |  |  |  |  |  |  |
| Constant | 3.40^*^  (0.21) | 3.77^*^  (0.26) | 3.14^*^  (0.24) | 3.45^*^  (0.24) | 3.40^*^  (0.23) | 3.59^*^  (0.24) |
|  |  |  |  |  |  |  |
|  | | | | | | |
| Observations | 938 | 1,047 | 1,031 | 1,020 | 1,016 | 1,011 |
| R^2^ | 0.38 | 0.35 | 0.32 | 0.25 | 0.26 | 0.39 |
| Adjusted R^2^ | 0.36 | 0.34 | 0.31 | 0.24 | 0.25 | 0.37 |
| F Statistic | 554.91^*^ | 551.24^*^ | 481.96^*^ | 332.35^*^ | 357.29^*^ | 624.80^*^ |

Unstandardized coefficient (standard error) reported. These are all random effects estimators

*p < .05 (2-tailed t-test)
