# Supplementary material for: Politics is making us sick: The negative impact of political engagement on public health during the Trump administration
Source: PLoS One. 2022 Jan 14;17(1):e0262022. doi: 10.1371/journal.pone.0262022 (PMC8759681; doi:10.1371/journal.pone.0262022)
Supplement: S9 Table — (DOCX) [file pone.0262022.s009.docx]

**Table S9.** Test of Fixed versus Random Effects Estimators

|  | Full Battery | Health  Scale | Emotional  Scale | Compulsion  Scale | Social Scale | Short Form  Scale |
| --- | --- | --- | --- | --- | --- | --- |
| Hausman test | .34, 2 df  P=.84 | .50, 2 df  P=.84 | 3.86, 2 df  P=.14 | 1.8, 2 df  P=.39 | .03, 2df  P=.98 | 1.74, 2df  P=.41 |

This table reports Hausman tests for each of the panel models. The alternative hypothesis is that one model (random or fixed effects) is inconsistent and the test statistic is distributed as a chi square. A significant difference indicates fixed effects model is preferred over random effects. Because this is a short panel (two time points) and many of the individual traits are time invariant the variance in the predictors is concentrated between rather than within subjects. Time invariant variables are dropped from such models because they zero out – the fixed effects estimator mathematically is a time demeaned average, so if there is no across variance the mean is equal to the observed value and subtracting the mean from the observed value equals zero. All these factors argue for using a random effects estimator.
